# Evaluation of medication errors in nursing during the COVID-19 pandemic and their relationship with shift work at teaching hospitals: a cross-sectional study in Iran

**DOI:** 10.3389/fmed.2023.1200686

**Published:** 2023-09-21

**Authors:** Abdolreza Gilavand, Negar Jafarian, Kourosh Zarea

**Affiliations:** ^1^Department of Medical Education, School of Medicine, Ahvaz Jundishapur University of Medical Sciences, Ahvaz, Iran; ^2^Department of Community Medicine, School of Medicine, Ahvaz Jundishapur University of Medical Sciences, Ahvaz, Iran; ^3^Nursing Care Research Center in Chronic Diseases, School of Nursing and Midwifery, Ahvaz Jundishapur University of Medical Sciences, Ahvaz, Iran

**Keywords:** medication errors, shift work, nursing, teaching hospitals, COVID-19

## Abstract

**Introduction:**

Medication errors in nursing negatively affect the quality of the provided health-treatment services and society’s mentality about the health system, threatening the patient’s life. Therefore, this study evaluates medication errors in nursing during the COVID-19 pandemic and their relationship with shift work at teaching hospitals.

**Materials and methods:**

All the nurses working at teaching hospitals affiliated with Ahvaz Jundishapur University of Medical Sciences (southwest of Iran) comprised the statistical population of this research (260 participants). Data were collected using three questionnaires: a demographic characteristics questionnaire, a medication error questionnaire, and the standard Circadian Type Inventory (CTI) for a normal physiological cycle.

**Results:**

At least one medication error was observed in 83.1% of nurses during their work span. A medication error was found in 36.2% of nurses during the COVID-19 pandemic (over the past year). Most medication errors (65.8%) occurred during the night shift. A significant relationship was detected between medication errors and shift work. Medicating one patient’s drug to another (28.84%) and giving the wrong dose of drugs (27.69) were the most common types of medication errors. The utmost medication error was reported in emergency wards. The fear of reporting (with an average of 33.06) was the most important reason for not reporting medication errors (*p* < 0.01).

**Discussion and conclusion:**

Most nurses experienced a history of medication errors, which were increased by shift work and the COVID-19 pandemic. Necessary plans are recommended to reduce the fatigue and anxiety of nurses and prevent their burnout, particularly in critical situations. Efforts to identify risky areas, setting up reporting systems and error reduction strategies can help to develop preventive medicine. On the other hand, since the quality of people’s lives is considered the standard of countries’ superiority, by clarifying medical errors, a higher level of health, satisfaction and safety of patients will be provided.

## Introduction

Patient safety is one of the significant dimensions of health care, and harming a patient or someone who seeks health conflicts with the philosophy of health care (1). The most widespread types of medical errors are medication errors, which are currently used as an indicator to determine the level of patient safety at hospitals because of their high incidence and possible risks for patients (2). Drug administration to patients is a complex process that requires the knowledge, decision-making, and correct practices of employees working in hospital wards. Medication errors may occur at any stage of the medication process (3). Lack of pharmacology knowledge, incorrect drug calculations, failure to follow the planned protocols, illegible handwriting of doctors, and similarities in the shape, packaging, and names of drugs are among the cases that have been shown to contribute to the occurrence of medication errors. However, issues such as lack of time, fatigue, inadequate personnel, and the absence or lack of equipment are also some of the hidden issues that are indirectly involved in medication errors (4). Achieving a global commitment to reduce the severity and prevent drug-related injuries by 50% within 5 years was one of the primary goals of WHO in 2017 (3, 5). A systematic review study aiming to identify the barriers to reporting medication errors among nurses has highlighted organizational barriers (inadequate reporting systems, management behavior, and unclear definition of medication error), and professional and personal barriers (fear of management/colleagues/litigation, personal reasons, and insufficient knowledge of errors) in this context (6). The complications of medication errors include injury to the patient, death of patients, lack of patients’ and their families trust in the health and treatment system, increase in hospital costs, professional damage to nursing, and threatening patient safety and their rights (7, 8). About 10% of the leading cause of death is associated with medication errors worldwide. In developed countries, about one out of 10 patients is injured while receiving hospital care, and half is preventable (6, 9, 10).

Work shift refers to any type of work accomplished regularly and specifically outside of the daily work time slot (7 am to 6 pm) (11). As with other living beings, humans have a rhythmic nature. One of the known rhythms in human physiology and psychology is circadian rhythms, which have a natural movement and show resistance to sudden changes in their routine and daily schedule. The function of this system is to prepare the brain and body for active sleep or awakening at specific times of the day. Therefore, changes in this system result in anxiety, irritability, bad mood, gastrointestinal disorders, reduced sleep hours, work efficiency, and safety performance (due to incoordination in the biological clock); these characteristics may cause considerable problems in shift workers (12–14). The medical education and healthcare systems are integrated in Iran. Therefore faculty members and students are also engaged in the treatment of patients at teaching hospitals in addition to teaching and research work (15). Not paying enough attention to the correct principles in medication can lead to ethical and professional problems threatening the patient’s life. Therefore, it is necessary to address this issue to prevent medication errors or find strategies to prevent such cases. This research focuses on issues mentioned above, considering that very few studies have so far been conducted on the level, type, and causes of medication errors at teaching hospitals during the COVID-19 pandemic. Accordingly, the present study aims to investigate the status of medication errors in nursing and their relationship with shift work at teaching hospitals during the COVID-19 pandemic.

## Materials and methods

The current descriptive, correlational, and cross-sectional study was include in 2021. The statistical population of this research included all nurses working at teaching hospitals of Ahvaz Jundishapur University of Medical Sciences (southwest of Iran). Using the convenience sampling method, a sample size of at least 200 individuals was determined according to a similar study (16). A total of 260 people participated in this research. The inclusion criteria consisted of employment as a nurse at a teaching hospital and their willingness to participate in this study. People who were reluctant to participate in the study and those who did not complete the questionnaires were excluded. In the current study, medication error is defined as any error in the medication process whether or not it has an adverse outcome. Medication errors were identified by clinical pharmacists using medication errors system under the supervision of medication safety officer. To comply with ethics in the research, all participants initially completed a written consent form to participate in the research. The consent form clearly stated that participants were free to decide to participate in the study and were not compelled to do so in any way or by anyone. In this study, data were collected using three questionnaires as follows:

1. A questionnaire for nurses’ demographic information.
2. A medication error questionnaire, modeled based on the standard questionnaire of Wakefield et al. (17) and a researcher-made questionnaire (16). This questionnaire contains four parts, the first of which includes some general questions about the job status of nurses. The second part consists of 10 questions about determining the type of medication errors (if present) in the past year. The third part contained questions about the causes of medication errors and the reasons for not reporting medication errors. The validity and reliability of this questionnaire were confirmed previously.
3. The standard Circadian Type Inventory (CTI) for a normal physiological cycle contains two independent factors. The first factor, called “Flexible/Rigid,” represents a stable circadian rhythm in the individual. A high score from this factor (> 18.75) indicates the person’s flexibility with the work shift system, and the person can stay awake during the night or the day on unusual days. The second factor, called Languid/Vigorous, represents the individual’s circadian rhythm range, and people obtaining a high score (> 22.75) can overcome their drowsiness with difficulty (18).

Categorical data were reported as percentages, frequencies, Mean, standard deviation. Level of statistical significance was declared at value of *p* < 0.05. The statistical analysis was carried out using IBM’s Statistical Package for the Social Sciences (SPSS) version 24.0 software (Chicago, IL, United States).

This research resulted from a medical student (GP) thesis in medicine and was approved by Ahvaz Jundishapur University of Medical Sciences. (No. U-00246& Ethics Code of IR.AJUMS.REC.1400.572).

## Results

This study was conducted on 260 nurses working at teaching hospitals during the COVID-19 pandemic. The age of these nurses averaged 29.46 years. There were 61.9 and 53.1% married and single nurses, respectively. For the education level, 80.8% of the nurses held a bachelor’s degree, and the rest were postgraduates. Regarding the number of shifts, 50.4 and 43.8% of the nurses worked in single and two shifts, respectively. Moreover, 43.4% of the nurses had work experiences of 1–5 years, and 43.8% worked in private centers simultaneously, with an average work experience of 2.12 in private centers. In terms of work shift status, 80.4, 17.7, and 1.9% worked in rotation, in the morning shift, and in the evening shift, respectively.

Table 1 represents the general status of nurses’ medication errors according to the dimensions of the questionnaire.

**Table 1:** Status of nurses’ medication errors.

Variable	Classification	Frequency	Frequency (%)
Have you performed medication errors during your work experience?	Yes	216	83.1
	No	44	16.9
Have you performed medication errors during the COVID-19 pandemic?	Yes	94	36.2
	No	166	63.8
Have you reported medication errors to your colleagues, supervisor, and nursing office?	Yes	58	61.71
	No	36	38.29
Have you witnessed the medication errors of other colleagues? Or have you been informed of such errors?	Yes	227	87.3
	No	33	12.7
The ward where you performed medication errors	Orthopedics	15	5.8
	Neonatal	10	3.8
	Emergency	108	41.5
	Surgery	64	24.6
	Internal	17	6.5
	Infectious	11	4.2
	ICU	35	13.5

Table 2 shows the time and number of nurses’ medication errors according to the dimensions of the questionnaire.

**Table 2:** The time and number of medication errors by nurses.

Variable	Classification	Frequency	Frequency (%)
In which shift did these medication errors occur most frequently?	Night	171	65.8
	Morning	5	1.9
	Morning and evening	73	28.1
	Evening	11	4.2
	No errors	78	30
	Wrong patient	3	1.15
	Wrong drug administration route	3	1.15
	Drug administration without a doctor’s prescription	2	0.8
	Drug exclusion from the patient’s drug list	10	3.84
	Medication at unscheduled hours	12	4.61
	Giving one patient’s drug to another one	75	28.84
	Medicating the wrong drug dose	72	27.69
	Incorrect infusion rate	5	1.92

Table 3 lists the factors affecting the incidence of medication errors among nurses according to the dimensions of the questionnaire.

**Table 3:** Factors affecting the incidence of medication errors by nurses.

Variable	Mean	SD	Min	Max
Nurse-related factors	17.78	3.15	12	27
Ward-related factors	21.62	2.95	10	28
Nursing management factors	25.59	2.24	19	29
Total scores of medication error reasons	64.99	5.21	48	78

Table 4 presents the reasons for not reporting medication errors by nurses according to the dimensions of the questionnaire.

**Table 4:** Reasons for not reporting medication errors by nurses.

Variable	Mean	SD	Min	Max
Fear of reporting consequences	33.06	7.12	22	52
Factors related to the reporting process	6.41	2.38	3	15
Fear of management factors	20.65	2.92	12	25
Total scores of the questionnaire for non-reporting reasons	60.13	8.67	446	79

Table 5 reports the descriptive indicators of the CTI dimensions according to the dimensions of the questionnaire.

**Table 5:** Descriptive Indicators of the CTI Dimensions.

Variable	Mean	SD	Min	Max
LV	18.53	3.19	11	28
FR	11.63	3.24	5	22
Total score	30.17	4.22	25	47

The frequency and frequency percentage of the FR classification of the CTI are shown in Table 6, indicating that 10 and 250 people are flexible and inflexible, respectively.

**Table 6:** The frequency and frequency percentage of the FR classification.

Variable	Classification	Frequency	Frequency (%)
FR classification	Flexible	10	3.8
	Inflexible	250	96.2

Table 7 shows a significant relationship between the medication error variable and shift work measured using the Chi-squared test (*p* < 0.001).

**Table 7:** The relationship between the medication error variable and work shifts.

Variable	Classification	Frequency	Frequency (%)	Frequency	Frequency (%)	Frequency	Frequency (%)	*P*
		Rotation		Morning		Evening		
Have you performed a medication error during work?	Yes	175	83.7	41	89.1	0	0	<0.001
	No	34	16.3	5	10.9	5	10.9	

## Discussion

The results of our study demonstrated that 83.1 and 36.2% of nurses experienced at least one medication error throughout their working period and during the COVID-19 pandemic (over the past year), respectively. Furthermore, medication errors mainly were performed during night shifts (65.8%), with a significant relationship between medication errors and work shifts. The most common type of medication error was giving one patient’s medication to another (28.84%) and prescribing a wrong dose of drugs (27.69). The utmost medication errors were reported in emergency wards. In addition, fear of the reporting consequences (with an average of 33.06) was the main reason for not reporting medication errors.

A high rate of medication error was reported in our study. In a study in Yazd City (the center of Iran), Bagheri et al. reported that 66.7% of nurses experienced medication errors, and 40.6% performed medication errors only once (19). In the study by Dashti et al. in the city of Ardabil in northwestern Iran, the incidence rate of at least one medication error in the nurses under study during the past 6 months was 86.4%, with an average of 3.6 errors per nurse (20). Alsulami et al. reviewed medication errors in 45 studies from 10 Middle Eastern countries and reported error rates from 7.1 to 90.5% (21). In another review study, Rababa’h et al. reported a high rate of medication errors (0.1–96%) in Jordan (22). Alyami et al. identified 4,860 medication errors in a Saudi Arabian hospital during the COVID-19 pandemic between 2018 and 2020 (23). Asensi-Vicente et al. reviewed 19 related articles and reported a high rate of medication errors among nursing students (24).

In our study, most medication errors (65.8%) occurred during the night shift, and there was a significant relationship between medication errors and work shifts. Working at night is contrary to human nature and circadian rhythms and causes disorders such as sleep disorder, fatigue, inattentiveness, lack of concentration, and excitability. Nurses and midwives account for approx - 50% of the global healthcare shift workforce. Work shift causes sleep disorder, fatigue, and adverse effects on nurses’ health and patient safety and care (25). Books et al. investigated shift work (night work shifts) and its effects on nurses’ health. They claimed that the risk of short sleep duration, family stress, and mood changes increased because of night shift work (26). During the COVID-19 pandemic, Lee et al. evaluated the prevalence of fatigue (62.0%), depression (52.1%), insomnia (20.7%), and daytime sleepiness of nurses (36.1%); insomnia and drowsiness were significantly associated with fatigue (27). Di Muzio et al. reviewed 19 eligible articles and denoted night shifts and the resulting short sleep duration as the primary cause of medication errors (28). In a study on the COVID-19 pandemic, Mohammed et al. reported a high percentage of medication errors among nurses in Ethiopian hospitals and mentioned night shift work as the leading cause of medication errors (29). Bagheri et al. presented evidence that 58.7% of medication errors occurred during the night shift in Yazd City, central Iran (19).

In the present study, most medication errors were reported in emergency wards. In Iran, Izadpanah et al. reported a medication error rate of 41.9 in emergency and neonatal wards within 1 month (30). Altogether, the findings of studies conducted on identifying error types occurring in emergency wards in Iran and other countries represent different types of errors, suggesting that medication errors can occur at any medication process stage. However, considerable differences can be expected in the type and number of reported errors due to differences in various healthcare and treatment systems in terms of the nurse–patient ratio, nurses’ work records and experiences, the number of prescribed drugs, the type of prescribed drugs, and the service provision method.

Our study showed that giving one patient’s medication to another and prescribing the wrong dose of medication were the most reported types of medication errors. These results correspond to those of Alyami et al. in Saudi Arabia during the COVID-19 pandemic between 2018 and 2020. In their study, ordering/prescribing/transcription, inappropriate dosage, dosage units, and medication repetition were the most common medication errors (23). In a review study, Alsulami et al. examined medication errors in Middle Eastern countries and reported wrong dosage and wrong frequency as the most common type of prescription errors (21). A review article by Rababa’h et al. indicates a high rate of medication errors in Jordan. Prescription errors were the most common error reported in 15 studies examined. The prevalence of prescription errors ranged from 0.1 to 96% (22).

In our study, the fear of reporting consequences (with an average) was the primary reason for not reporting medication errors. In similar studies, the fear of reporting consequences, including the reduction of evaluation scores, disciplinary punishment, and involvement with legal issues, was the highest reason for not reporting medication errors (7, 31–35). An important issue is a need to establish effective communication between nursing administrators and nurses so that people can follow the principles of professional ethics and report mistakes without concerns about the consequences of their false reporting.

In a review study, Aljabari and Kadhim examined 30 eligible articles regarding the barriers to reporting medical errors. Based on the results, the fear of consequences was announced as the main barrier to reporting (63%) (33).

### Limitations

Despite all the researchers’ efforts to investigate the status of nurses’ medication errors in the Covid-19 pandemic, they believe that this research may not have investigated all aspects of nurses’ medication errors. Because without a doubt, the long-term effects and destructive consequences of this long-term epidemic require further investigation in the future.

## Conclusion

Our study demonstrated that most of the nurses experienced a history of medication errors, which were aggravated by shift work and the COVID-19 pandemic. However, considerable differences in the type and number of reported errors may be expected because of the differences existing in various health and treatment systems in terms of the nurse–patient ratio, nurses’ work records and experiences, the number and type of prescribed drugs, and service provision procedures. The medication errors of nurses negatively influence the quality of provided health and treatment services and society’s mentality about the health system as well as on patient safety and care. Therefore, it is recommended to reduce the fatigue and anxiety of nurses and prevent their burnout, particularly in critical situations, using necessary planning. Furthermore, the educational content should be enriched, and more substantial supervision and management should be established on the work of medical personnel. Efforts to identify risky areas, setting up reporting systems and error reduction strategies can help to develop preventive medicine. On the other hand, since the quality of people’s lives is considered the standard of countries’ superiority, by clarifying medical errors, a higher level of health, satisfaction and safety of patients will be provided.

## Data availability statement

The original contributions presented in the study are included in the article/supplementary material, further inquiries can be directed to the corresponding author.

## Ethics statement

This research was approved by Ahvaz Jundishapur University of Medical Sciences. (No. U-00246& Ethics Code of IR.AJUMS.REC.1400.572). All participants initially completed a written consent form to participate in the research.

## Author contributions

AG: designed research, conducted research, wrote manuscript, and primary responsibility for final content. NJ: analyzed data. KZ: review and editing. All authors contributed to the article and approved the submitted version.
